# Relationship between parenthood and cortical thickness in late adulthood

**DOI:** 10.1371/journal.pone.0236031

**Published:** 2020-07-28

**Authors:** Edwina R. Orchard, Phillip G. D. Ward, Francesco Sforazzini, Elsdon Storey, Gary F. Egan, Sharna D. Jamadar

**Affiliations:** 1 Turner Institute for Brain and Mental Health, Monash University, Melbourne, Australia; 2 Monash Biomedical Imaging, Monash University, Melbourne, Australia; 3 Australian Research Council Centre of Excellence for Integrative Brain Function, Australia; 4 Department of Epidemiology and Preventive Medicine, Monash University, Melbourne, Australia; 5 Department of Neuroscience (Medicine), Monash University, Alfred Hospital Campus, Melbourne, Victoria, Australia; Nathan S Kline Institute, UNITED STATES

## Abstract

Pregnancy and the early postpartum period alter the structure of the brain; particularly in regions related to parental care. However, the enduring effects of this period on human brain structure and cognition in late life is unknown. Here we use magnetic resonance imaging to examine differences in cortical thickness related to parenthood in late life, for both sexes. In 235 healthy older women, we find a positive relationship between parity (number of children parented) and memory performance in mothers. Parity was also associated with differences in cortical thickness in women in the parahippocampus, precuneus, cuneus and pericalcarine sulcus. We also compared non-parents to parents of one child, in a sub-sample of older women (*N* = 45) and men (*N* = 35). For females, six regions differed in cortical thickness between parents and non-parents; these regions were consistent with those seen earlier in life in previous studies. For males, five regions differed in cortical thickness between parents and non-parents. We are first to reveal parenthood-related brain differences in late-life; our results are consistent with previously identified areas that are altered during pregnancy and the postpartum period. This study provides preliminary evidence to suggest that neural changes associated with early stages of parenthood persist into older age, and for women, may be related to marginally better cognitive outcomes.

## Introduction

New parents face an endless series of novel challenges to ensure the survival of their offspring. In addition to their own personal needs and existing responsibilities, new parents must confront persistent demands for care and protection of their infant. As children grow, new challenges arise: nappies and baby bottles are swapped for school lunches and soccer practice. The increased environmental complexity of parenthood continues for years—even decades—and is increased by each subsequent child; requiring simultaneous care across different stages of dependency. Successfully navigating these challenges requires rapid behavioural change and skill acquisition, driven by a combination of neuroendocrine and experiential factors [1]. The lasting impact of parenthood on the human brain is unknown, with research in this field still in its infancy.

The majority of existing studies of the parental brain are in preclinical models, and almost all have been conducted in female animals. Converging evidence from non-human models suggests that maternal behaviours are related to neural adaptations, including changes to dendritic morphology, cell proliferation and gene expression [2, 3]. Although well established in rodents, the impact of reproductive experience on the human brain is less clear. Existing studies on human parenthood tend to be infant-centric rather than parent-centric; in other words, most studies focus on the parents’ response to the infant, rather than more generalised changes to the parental brain. Few studies have examined structural changes associated with parenthood in humans, of which most have focused on changes in females but not males. Sample sizes tend to be small (between 8–25 women), which may explain the inconsistent patterns of results. Oatridge et al. found that during pregnancy, total brain size decreased and ventricular volume increased compared with pre-conception levels, and returned to pre-conception size by 24 weeks post-partum [4]. Kim et al. found that new mothers showed increased grey matter volumes throughout the cerebral cortex four months post-partum, compared with 1-month post-partum [5]. Increased grey matter volume in the midbrain at four months post-partum predicted the mother’s positive opinions of her own baby. In a follow-up study, Kim, Dufford, & Tribble [6] found increasing grey matter thickness in mothers across the first 6 months post-partum, and identified a positive correlation between cortical thickness and parental self-efficacy. Conversely, Hoekzema et al. showed that pregnancy resulted in substantial *decreases* in grey matter volume, particularly in networks subserving social cognition and theory of mind [7]. Reduced grey matter in mothers across pregnancy predicted increased mother-infant attachment and was sustained for at least two years post-partum. Preliminary evidence also suggests that fatherhood modulates plasticity in regions related to parental care. Kim et al. [8] found regional grey matter volume changes (both increases and decreases) in fathers at four months post-partum compared with one month post-partum. Together, the results of these studies show a pattern of increased and decreased grey matter in the early stages of parenthood (pregnancy and the first postpartum years). Longitudinal studies have shown a pattern of structural plasticity in the postpartum, consistent with decreased grey matter across pregnancy and increased grey matter in the postpartum period [4, 9]. This suggests that early parenthood involves dynamic and stage-specific neural re-organisation, and contains periods of cortical thinning (pregnancy) and thickening (postpartum months). Consequently, the neurobiological effects of parenthood on the human brain may be subtle and require larger studies to characterise accurately.

Evidence from preclinical models suggest that the changes may be permanent. Strikingly, preclinical evidence suggests that motherhood may convey a protective effect against dementia. Gatewood et al. found reduced hippocampal amyloid deposits and attenuated memory decline in older mother rats, compared with older virgin rats [10]. Thus, reproductive experience appears to benefit rodents into senescence, with hormonal and environmental changes that interact to produce a parental brain that is healthier, more flexible and resistant to age-related decline [10, 11]. Determining whether these neuroprotective effects are evident in humans, as well as rodents [12] and other mammals (e.g. primates [13]), will contribute to our understanding of the protective advantages of parenthood in late-life. In humans, it is not yet clear how long parenthood-related changes to the brain are sustained after the early parental period. However, it appears the neural effects of parenthood persist at least into mid-life. In a large middle-aged sample (*N* = 12,021), de Lange et al. [14] found less evidence of brain ageing in mothers compared to non-mothers, and a relationship between number of children and “younger looking” brains, suggesting a neuroprotective effect of motherhood on *brain-age*. This evidence of structural neuroprotection of parenthood has also been found in males [15]. In addition, middle-aged parents in the UK Biobank have faster response times and fewer errors on visual memory tasks than childless men and women [15]. This suggests that the neural changes associated with parenthood in humans are long-lasting, and potentially influential for brain ageing in late-life.

In the present study, we investigated the relationship between parenthood and the structure of the human brain in late life. Here, we examine the ‘dose’ effects of parenthood by examining the relationship between cortical thickness and the number of children a person has parented. We used data from a large healthy aged sample that forms part of the ASpirin in Reducing Events in the Elderly (ASPREE) clinical trial; specifically, the ASPREE-NEURO sub-study [16]. We also examine late-life differences in brain structure between parents and non-parents. We hypothesised that (1) regional cortical thickness will differ between parents and non-parents and (2) be associated with the number of children a person has parented. We expected to find a relationship between cortical thickness and parenthood for both sexes. However, since females have a larger parenthood-related hormonal burden during pregnancy; and demographically, women tended to be primary caregivers in this aged cohort, we anticipated that changes found in the maternal brain would be evident in the paternal brain to a lesser extent [17].

## Methods

We used data that formed part of the ASPREE-NEURO study, a sub-study of the ASPirin in Reducing Events in the Elderly (ASPREE) clinical trial (Baseline characteristics of the full ASPREE sample are reported in McNeil et al. [18]). All methods were approved by the Monash University Human Research Ethics Committee, in accordance with the Australian National Statement on Ethical Conduct in Human Research (2007). The data that support the findings of this study are available from ASPREE International Investigator Group, but restrictions apply to the availability of these data, which were used under license for the current study. Data are however available from the authors upon request and with permission of ASPREE International Investigator Group (https://aspree.org).

### Participants

Imaging data were acquired as part of the ASPREE- NEURO sub-study [16], which aimed to determine the effect of low-dose aspirin on a range of MRI biomarkers (cerebral microbleeds, white matter hyperintensities) over a three-year period, in neurotypical adults aged 70-yrs and over. The ASPREE clinical trial was double-blinded, with approximately half of the participants taking aspirin, and half taking placebo. Analysis of the main study aims is in progress, and information about which participants were allocated to each group is not yet unblinded [18]. We included data from baseline (prior to study medication) to test the main hypotheses. We used the data from Year 1 and 3 (after randomisation to study medication) to test the reproducibility of the main findings (see Data Analysis section).

Healthy older adults in Australia were eligible to participate if aged 70-years and over, had no history of occlusive vascular disease, atrial fibrillation, cognitive impairment or disability; were not taking antithrombotic therapy, and did not have anaemia or high risk of major bleeding, or a diagnosis likely to cause death within five years. At study entry, each participant had a Modified Mini Mental Status Examination (3MS; [19]) score of at least 78/100, and were able to perform all six of the Katz activities of daily living (ADLS; [20]).

Participants included here (N = 573) were aged 70–88 years. Data from 21 participants contained incomplete MRI scans and were discarded. Data from five participants were excluded due to the presence of neurological conditions (history of brain cancer (n = 1), Parkinson’s disease (n = 4)). In the final sample, data from 547 participants aged 70–88 years (mean age [+/-stdev] = 73.9+/-3.5years, 89.06% right-handed) were retained, including n = 287 males (74.0 ±3.6 years), and n = 260 females (73.7 ±3.4 years). The study attrition is given in S1 Table in S1 File, and shows a 15% attrition across 3 years.

As part of a health outcomes questionnaire, ASPREE-NEURO participants were asked “How many children do you have?”. More detailed parenthood data (e.g. biological/adopted children, grandparenthood, primary/secondary caregiver etc.) were not collected. The number of children parented by males and females in our sample is shown in S1 Fig in S1 File.

### Data acquisition

#### Cognitive tasks

Participants completed a 30-minute battery of cognitive tasks, including the Controlled Oral Word Association Test (COWAT; [21]), Hopkins Verbal Learning Test- Revised (HVLT-R; [22]), Symbol Digit Modalities Test (SDMT; [23]), Stroop Test (Victoria version; [24]) and Center for Epidemiological Studies Depression Inventory (CES-D; [25]). This cognitive battery was completed once at the first timepoint (baseline).

#### Image acquisition and preparation

All MRI scans were obtained on a 3T Siemens (Erlangen, Germany) Skyra MR scanner at Monash Biomedical Imaging, Australia. ASPREE-NEURO sequence protocols are described in detail elsewhere [16]. Here, we used T1 and T2 data. T1-weighted magnetization-prepared rapid gradient-echo (MPRAGE) images were acquired (TR = 2300 ms, TE = 2.13 ms, TI = 900 ms, matrix size = 256x240x192, bandwidth = 230 Hz/pixel). T2-Fluid attenuated inversion recovery (FLAIR) images (TR = 5000ms, TE = 395ms, TI = 1800ms, matrix size = 256x240x160, bandwidth = 781Hz/pixel) were also acquired, and were used to support the performance of cortical parcellation.

To extract cortical thickness values, MR images were segmented using the ‘recon-all’ function of FreeSurfer v5.3.0 [26]. To optimise the extraction of the pial surface (cerebrospinal fluid-grey matter boundary), both T1 MPRAGE and T2 FLAIR scans were used. To control for MRI quality, scans were checked for outliers (2 standard deviations above or below the mean) on one or more measure (total volume, total white matter volume, total grey matter volume, cortical grey matter volume, subcortical grey matter volume). The FreeSurfer segmentations were then visually inspected by an independent rater, blind to study aims, subject sex, and number of children, for quality control and scans with poor segmentation were reprocessed and subsequently excluded if the quality was not improved. Cortical thickness values were extracted for 34 regions (plus mean thickness) for each hemisphere using the Desikan-Killiany atlas, producing 70 cortical thickness values in total (2 hemispheres x 34 regions + 2 x hemisphere means; [27]).

### Data analysis

A regression was conducted to control for the effects of age, education, systolic and diastolic blood pressure, BMI, cholesterol, and the Euler characteristic (a FreeSurfer-derived measure of data quality; [28]). These covariates were selected based on previous studies reporting correlations between these factors and cortical thickness [28–30], and the expectation of a relationship between education and parity. All subsequent analyses used the Z-scores of these residuals, and were non-parametric as number of children is discrete, ordinal data, with a non-normal distribution.

The ‘dose’ effect of the number of children on cortical thickness was tested using Spearman’s correlation (two-tailed). The analysis was performed on all 70 thickness measures for 504 older adult participants, n = 269 male (74.0 ±3.5 years) and 235 female (73.8±3.5 years) participants who had one or more children. To test the hypothesis that cortical thickness would differ between parents and non-parents, for both sexes, the cortical thickness of 70 brain regions were compared between individuals with one child and individuals with no children in 80 older adult participants, including males (n = 35; 73.4 ±3.7 years) and females (n = 46; 72.8 ±3.3 years). The effect sizes were quantified using Cohen’s *d*. Significance was estimated using a permutation test (10,000 permutations of group assignments).

Data analysis for the cognitive tasks mirrored the analysis structure of the MRI data. All cognitive tasks (COWAT, HVLT-R, SDMT, Color Trails, Stroop and CES-D) were compared between participants who were childless (non-parents) and those who were parents to one child. The relationship between all cognitive tasks and number of children was also examined for participants with more than one child.

Note that we did not apply Bonferroni correction to our results. However, given the large number of comparisons in this study, we examined the reproducibility of the findings across timepoints within our data set to identify effects worthy of future study. Participants were scanned at three timepoints, at baseline (prior to administration of study medication), Year 1 and Year 3. We tested our hypotheses with data from Timepoint 1 (prior to aspirin exposure) and then tested the reproducibility of our MRI results by examining if they were maintained at Timepoints 2 and 3. The effect of parenthood in late life is not expected to change across the three-year measurement period; rather, any differences in the relationship between parenthood and cortical thickness across timepoints should be largely attributable to measurement error. Note, that this approach tests for false positives due to measurement error, it does not control for false negatives. We report the main results for Timepoint 1 in the Results section; the reproducibility results are reported in S2 and S3 Tables in S1 File. Whilst we acknowledge the importance of reducing the inflation of type I errors, given the novelty of the research question, it is also important to identify potential effects for future studies, so that hypotheses can be formed for specific regions in replication studies.

## Results

Descriptive statistics and demographic variables are given separately for females and males and each analysis in Table 1.

**Table 1:** Demographic statistics and sample sizes for females and males for each analysis.

		Females	Males
		Mean (SD)	Mean (SD)
**Total**		N = 260	N = 287
	Age	73.74 (3.43)	74.00 (3.56)
	Education	3.46 (1.75)	3.62 (1.68)
**Nulliparous**		N = 25	N = 17
(0 Children)	Age	72.55 (2.33)	72.55 (2.87)
	Education	4.36 (1.80)	3.72 (1.75)
**Primiparous**		N = 20	N = 18
(1 Child)	Age	73.12 (4.13)	74.06 (4.01)
	Education	3.57 (1.83)	3.48 (1.60)
**Multiparous**		N = 235	N = 269
(1 or more Child)	Age	73.86 (3.50)	74.12 (3.61)
	Education	3.37 (1.72)	3.63 (1.68)

Data for the correlation analyses used multiparous participants (those with one or more child) and the analyses comparing parents to non-parents used the data from the nulliparous and primiparous participants. Education is measured in levels (1 = less than 9 years, 2 = 9–11 years, 3 = 12 years, 4 = 13–15 years, 5 = 16 years, 6 = 17–21 years).

**Fig 1:**
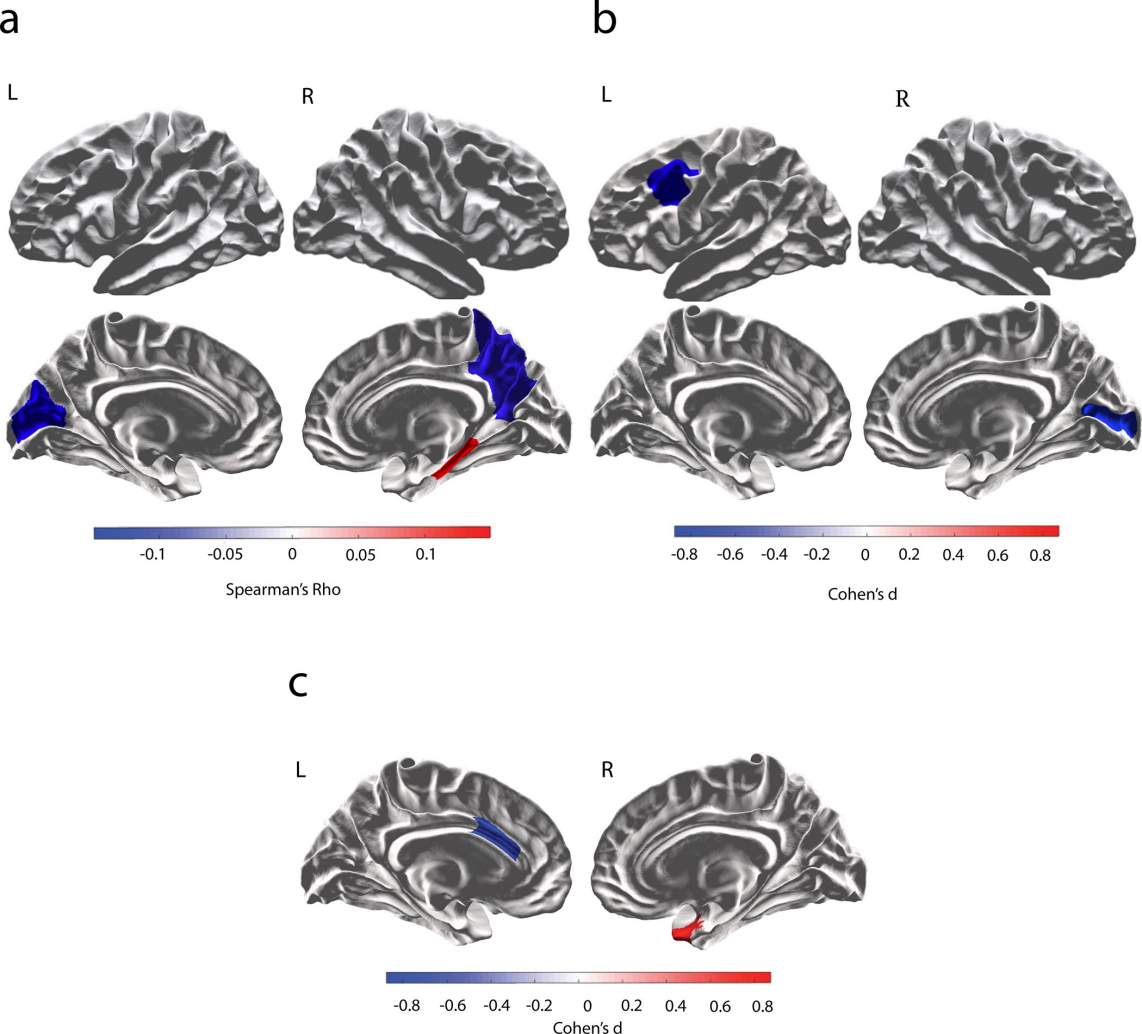
(a) Spearman’s Rho for brain regions showing a significant relationship between cortical thickness and number of children for female. Regions highlighted in red (right parahippocampal gyrus) depict a positive relationship, regions highlighted in blue (left pericalcarine sulcus and cuneus) depict a negative relationship. (b) Cohen’s d for the difference between females with one child and females with zero children. Regions highlighted in blue (left caudal middle frontal/DLPFC, right pericalcarine sulcus) depict a negative Cohen’s d and thinner GM in mothers, compared with non-mothers. (c) Cohen’s d for the difference between males with one child, to males with zero children. Regions highlighted in red (right temporal pole) depict a positive Cohen’s d and thicker GM in fathers, regions highlighted in blue (left caudal anterior cingulate cortex) depict a negative Cohen’s d and thinner GM in fathers compared with nonfathers.

For mothers, three regions showed a significant relationship with number of children (Table 2; Fig 1A). Cortical thickness in the left pericalcarine sulcus (*Spearman’s Rho* = -0.10, *p* = .11), left cuneus (*Spearman’s Rho* = -0.13, *p* = .054) and right precuneus (*Spearman’s Rho* = -0.12, *p* = .073) decreased as the number of children parented increased. Conversely, the cortical thickness of the right parahippocampal gyrus (*Spearman’s Rho* = 0.15, *p* = .026) increased with the number of children parented (Fig 1A). Each of these results were reproduced across timepoints (S2 Table in S1 File).

**Table 2:** Relationship between number of children and cortical thickness for females.

Region	Hemisphere		Time-point 1
			N = 235
Parahippocampal Gyrus	Right	*Rho (Spearman)*	0.15
		*p*-value	0.026
Cuneus	Left	*Rho (Spearman)*	-0.13
		*p*-value	0.054
Pericalcarine Sulcus	Left	*Rho (Spearman)*	-0.10
		*p*-value	0.113
Precuneus	Right	*Rho (Spearman)*	-0.12
		*p*-value	0.073

In females, two regions showed consistent differences in cortical thickness between mothers of one child and non-mothers (Table 3; Fig 1B). The left dorsolateral prefrontal cortex (*d* = -.92, *p* = .006) and the right pericalcarine sulcus (*d* = -.59 *p* = .065) showed thinner cortex in mothers compared with non-mothers. These results were retained across all three timepoints.

**Table 3:** Cortical thickness differences between parents (one child) and non-parents for males and females.

**Females**	**Region**	**Hemisphere**		**Time-point 1**
				N_(Mothers)_ = 20; N(_Non-Mothers)_ = 25
	Caudal Middle Frontal Gyrus	Left	*Cohen’s d*	-0.92
			*p*-value	0.006
	Pericalcarine Sulcus	Right	*Cohen’s d*	-0.59
			*p*-value	0.065
**Males**	**Region**	**Hemisphere**		**Time-point 1** N_(Fathers)_ = 18; N(_Non-Fathers)_ = 17
	Caudal Anterior Cingulate Cortex	Left	*Cohen’s d*	-0.69
			*p*-value	0.053
	Temporal Pole	Right	*Cohen’s d*	1.00
			*p*-value	0.007

In males, two regions showed significant differences in cortical thickness between fathers of one child and those who had not fathered children (Fig 1C). The left anterior cingulate (Cohen’s *d* = -.69, *p* = .053) showed thinner grey matter in fathers compared with non-fathers, and the right temporal pole (Cohen’s *d* = 1.00, *p* = .007) showed thicker grey matter in fathers compared with non-fathers. While the trend in the anterior cingulate was retained across the three timepoints, the effect in the temporal pole was notably weaker across timepoints (from *d* = 1.00 to 0.21; S3 Table in S1 File). As such, the effect in the temporal pole should be interpreted with caution.

The location and direction of these results are compared to grey matter changes found across early parenthood (pregnancy and the postpartum period) in Table 4.

**Table 4:** Present results in the context of current literature: Grey matter changes observed across early parenthood (pregnancy and the postpartum period). GM = grey matter, ↑ = increased, ↑ = decreased, R = right, L = left.

Present Results				Hoekzema et al. 2017	Luders et al., 2020	Kim et al. 2010	Lifosky et al., 2019	Kim et al. 2018	Kim et al. 2014
Cortical Thickness in Late-Life Parenthood									
Region (Hemisphere)				Volume	Volume	Volume	Volume	Thickness	Volume
				Mothers v Non-Mothers	Mothers	Mothers	Mothers v Non-Mothers	Mothers	Fathers
				Pre-pregnancy & 2.5mth postpartum	1 & 6 weeks postpartum	1 & 4 months postpartum	2 & 5 months postpartum	<6 months postpartum	1 & 4 months postpartum
**Females**	Number of Children Mothered	Parahippocampal Gyrus (R)	↑GM	↓GM		↑GM			
		Precuneus (R)	↓GM	↓GM	↑GM	↑GM			↓GM
		Cuneus (L)	↓GM		↑GM			↑GM	
		Pericalcarine Sulcus (L)	↓GM		↑GM			↑GM	
	Mothers v Non-Mothers	Pericalcarine Sulcus (R)	↓GM						
		Middle Frontal Gyrus (L)	↓GM	↓GM	↑GM	↑GM	↑GM	↑GM	↓GM
**Males**	Fathers v Non-Fathers	Anterior Cingulate Cortex (L)	↓GM	↓GM		↑GM	↑GM		
		Temporal Pole (R)	↑GM						↑GM

**Fig 2:**
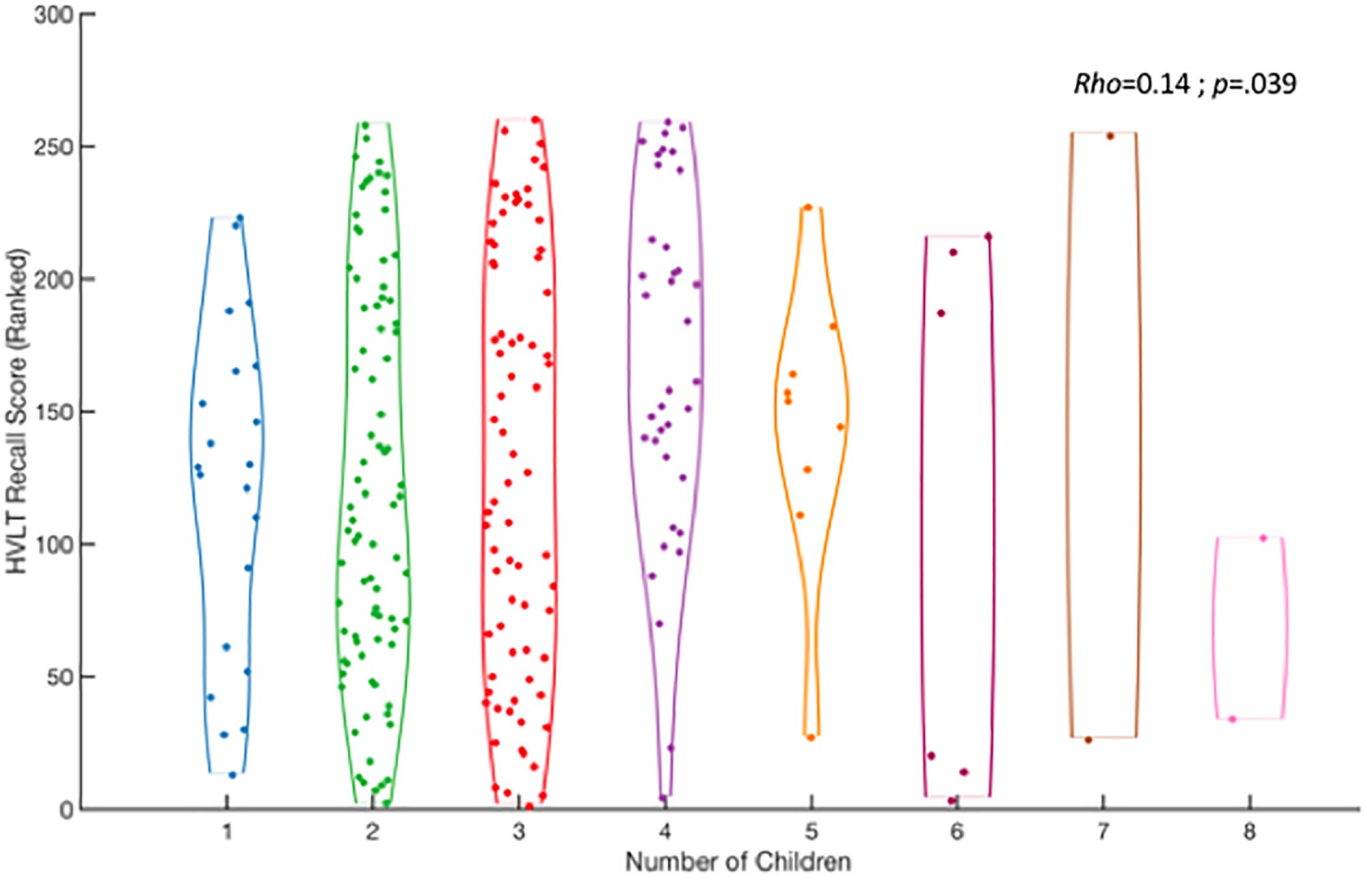
Relationship between Hopkins Verbal Learning Task (HVLT) recall score and the number of children a woman has mothered. HVLT score was first controlled for age and education, then converted to rank, such that each participant’s memory score (N = 260) was converted into rank order (1 to 260).

Females showed a marginally significant positive relationship between parity and delayed recall scores on the Hopkins Verbal Learning Test—Revised (HVLT-R), adjusted for age and education, such that women with more children showed better HVLT-R memory outcomes (Fig 2). There were no significant differences in cognition between mothers (of one child) and non-mothers. In males, no relationship was found between any cognitive test and parenthood.

## Discussion

Our results suggest that, while modest, changes in cortical thickness associated with parenthood are present in late life. No previous study has examined the effect of parenthood on brain structure in humans in late-life. The anatomical regions implicated in parenthood here are consistent with those previously found in human pregnancy and the early post-partum period in structural [5–9, 31] and functional (see Swain et al. [32] for review) studies. While this preliminary study was exploratory, we found that our results were reproduced across timepoints in this sample of healthy aged individuals (See S2 and S3 Tables in S1 File). Our results suggest that these effects are worthy of further study.

### Motherhood and cortical thickness

We found a positive relationship between number of children parented and cortical thickness in the parahippocampal gyrus of older adult mothers. This result is consistent with findings in early stages of parenthood, where grey matter changes are seen in the parahippocampal gyrus during pregnancy [7] and the postpartum period [5]. The parahippocampal gyrus is involved in memory consolidation and cohesion [33] and mediates cortico-hippocampal communication [34], and atrophy in the parahippocampal gyrus plays a key role in age-related memory decline [34]. A positive association between number of children parented and cortical thickness in a region associated with memory is compatible with our cognitive results, where memory outcomes were improved in older adult women with more children. Parenthood appears to be associated with a small but reliable improvement in memory outcomes for older mothers. This result is consistent with evidence from early stages of parenthood, where memory is impaired during pregnancy [35], returns to pre-pregnancy levels in the postpartum period [36], and, at least in rodents, is enhanced in late parenthood [10].

Our result is consistent with results seen in preclinical models. Spatial learning and memory are improved in female rodents with reproductive experience, and this effect is stronger in mothers with more than one pregnancy [37]. This cognitive benefit persists into late-life for parous female rats, who have increased hippocampal long-term potentiation, enhanced memory capacities and fewer signs of brain aging compared with aged nulliparous females; an effect that is enhanced with multiparity [10]. Our findings, that both memory performance and parahippocampal thickness increase with the number of children parented in female humans, are consistent with these reports and suggest that parenthood is positively related to human cognition in late life. We found no difference in cognitive performance between older mothers with one child (*N* = 20), and older women with no children (*N* = 25). It is possible that these effects would be observed using a larger sample size, or that memory ability is only linked to the cumulative hormonal and environmental changes associated with mothering a number of children.

Compared with childless women, those with one child showed thinner grey matter in the left dorsolateral prefrontal cortex. The dorsolateral prefrontal cortex is involved in high-order cognitive functions including executive functioning, cognitive control and emotional regulation [38]. Neuroimaging data highlights the importance of the prefrontal cortex in parenting behaviours, as this region is activated in almost every fMRI study of human mothers’ responses to infant stimuli [32]. Increased brain activity in a mother’s prefrontal cortex in response to her own baby compared to an unfamiliar baby, is positively associated with maternal sensitivity and higher quality of mother-infant interactions [39]. Conversely, decreased fMRI activation in the prefrontal cortex is correlated with difficulties adjusting to parenthood [40]. Neural changes in early stages of motherhood are likely to have evolved to assist in the dual survival of parent and young [3]. Three recent studies have shown maternal grey matter thickness [6] and volume [5, 7] changes in the prefrontal cortex across pregnancy and the early post-partum period. In all three of these studies, structural change was associated with adaptive maternal factors (increased attachment; higher parental self-efficacy; more positive opinions about their baby). Our results indicate that parenthood-related changes in the cortical thickness of the dorsolateral prefrontal cortex in mothers are present in late life.

Our results show thinner cortical thickness of the pericalcarine sulcus and cuneus for females. These results appear in both hemispheres across both analyses (childless versus one child, and number of children). This finding is intriguing, as it is not usually a region associated with age-related decline (e.g., [41]). Interactions with children provide a rich array of sensory experiences for mothers. Interactions involving multi-sensory cues (e.g., visual, auditory, tactile) between mothers and infants throughout the postpartum period, and continuing into late-parenthood, may alter the structure and function of the maternal sensory regions over time [6]. In female rodents, neural reorganisation of the olfactory bulb, parietal lobe, and somatosensory cortex were associated with the expression of maternal behaviours during the postpartum period [42]. This result may therefore suggest that sensory changes that occur in the postpartum period that serve to enhance maternal behaviours may be still present in older age.

### Fatherhood and cortical thickness

Compared with childless men, fathers showed thinner grey matter in the left anterior cingulate cortex and thicker grey matter in the right temporal pole. The effect in the temporal pole was notably weaker across timepoints, and so this effect may be a false positive result. These regions are involved in social cognition, including emotional regulation, empathy and theory of mind [43, 44]. Social cognition is a hallmark of the human parental brain, especially relevant for pre-verbal infants [45]. Theory of mind abilities, including empathy, contribute to successful social interactions and are important for human survival. Our results are consistent with previous studies in early stages of parenthood that found grey matter volume changes in the anterior cingulate cortex associated with early parenthood and fathers [8], and suggests they may persist into late life.

### Parenthood and cortical thickness in late life

We found modest but reproducible relationships between parenthood and cortical thickness in late life. As expected, the results were smaller in males than in females. We argue that these late-life structural brain differences associated with parenthood are related to the environmental complexity of parenthood. While the drastic hormonal shifts of pregnancy may play an initial role in mothers, the hormonal milieu of pregnancy returns to a pre-pregnancy state following parturition, at which time many hormonal and biological changes are resolved, i.e., uterus size, lactation, etc [46]. In our sample of older adults, the hormonal exposure of pregnancy occurred three or more decades earlier. In contrast, the environmental complexity of parenthood endures throughout the entire parenting experience, possibly throughout the life-span of a parent, and compounds with increasing number of children. Indeed, it is known from preclinical models that the mere presence of offspring is sufficient to produce neural changes associated with parenting, even in the absence of pregnancy-related hormones [47]. Child-rearing provides parents of both sexes with rich sensory stimulation and increased environmental complexity, with new parents required to engage in novel tasks and challenges [48]. This is consistent with the conceptualisation of caregiving as a dynamic, learning experience in humans, and as a period of plasticity [48, 49]. As such, parenthood is a continuum of experience, and may represent a learning environment that is sustained for two or more decades of an individual’s life [49]. Parenthood may therefore contribute to a person’s cognitive reserve, by increasing the cognitive and social demands of the individual.

In the present study, cortical thickness differences between parents and non-parents were evident in both sexes. This novel finding implicates the parental environment, which exists for both sexes. The analysis of cortical thickness differences between parents and non-parents included smaller sample sizes than the multiparity analyses, and this should be taken into account when evaluating the results. The neuroanatomical changes associated with parenting multiple children were only apparent for mothers, with no significant relationship identified in fathers. This may reflect differences in care-giving responsibilities between the sexes: the cohort examined in this study most likely included participants who were predominantly in ‘traditional’ care-giving arrangements, with roles and responsibilities as primary care-giving mothers and ‘bread-winning’ fathers [50]. The social structure of this generation may have limited the cumulative impact of parenthood for the males in this cohort. Therefore, the different effects observed in females and males may reflect the likely primary and secondary caregiver status of our participants, respectively, rather than the distinction between motherhood and fatherhood. Similar studies in future generations, with a presumably larger proportion of primary care-giving fathers, may see results that instead scale with time spent in care, rather than sex of the parent.

The diverse and subtle changes in cortical grey matter thickness measured in this study point to a rich and multi-layered process of cortical plasticity that begins during pregnancy (in females) and the early post-partum period (males and females), and is evident across the lifespan. It is important to note that we have observed both positive and negative associations with cortical thickness in relation to parenthood for both males and females. These regions have also shown grey matter changes across pregnancy and the early postpartum period (Table 4). In early parenthood, these regions have shown both increases [4–6, 8, 9, 31] and decreases [7, 8] in grey matter thickness or volume associated with measures of positive parental care (e.g. attachment, self-efficacy, positive opinions of baby). Generally, decreasing grey matter thickness is understood as neurodegeneration (atrophy) in ageing brains (e.g., [51]). However, in relation to parenthood, reductions in grey matter have been explained as a ‘fine-tuning’, similar to what we see in puberty [7]. Indeed, the morphometric changes (surface area, cortical thickness) of pregnancy highly resemble those occurring during adolescence, and decreased grey matter may reflect processes of synaptic pruning and myelination [52]. The physiological processes that can alter cortical thickness include changes in the number of synapses, glial cells or neurons, morphological changes to dendritic structure and axonal myelination, and vasculature changes—including angiogenesis—that influence blood volume and perfusion. While the study design precludes identification of the neurobiological mechanisms that underpin the observations, the novelty and social importance of the findings highlight the need for further studies: *in vivo*, to examine the microstructural, functional and neurovascular characteristics of parenthood; and post-mortem stereological, to determine the cellular and morphological origins of the cortical thickness changes. Without studies like these, it is very difficult to determine whether increases and/or decreases in grey matter thickness are adaptive for the late life brain in relation to parenthood.

The relationships between number of children parented and cortical thickness and memory performance found for mothers are potentially non-linear. In the current study, the relationship was obtained using Spearman’s correlation, which does not assume linearity, and instead measures the strength and direction of a monotonic relationship. A non-linear relationship between brain structure and number of children parented has been shown in a large sample of middle-aged women [14]. However, this non-linearity was not replicated in their follow-up study, showing region-specific effects in the same sample which were linearly related to the number of children parented [53]. Future studies should explore the potential for non-linear associations between parenthood and brain structure in middle- and late-life.

The data used in this study were acquired as part of a larger study with broader aims. Detailed demographic information related to parenthood was not obtained from the participants, and so information about the parenting experiences is not available. Many of these unknown factors, such as birth and feeding methods, hormone levels, parental sensitivity, and involvement with child-rearing (e.g. primary or secondary caregiver, living or not living with children, adoption, and so on) may also potentially impact maternal neuroplasticity [54]. We were also unable to control for pregnancies that did not result in live birth and/or parenthood experience (miscarriage, termination, still-birth, infant loss, or adoption). Therefore, we are not able to guarantee that some of our participants didn’t experience additional exposure to pregnancy hormones. Furthermore, it is important to note that reproductive experience is related to other traits which impact brain health in human ageing, including exposure to sex steroid hormones. Some of these traits are heritable (e.g. age at menarche and menopause) and common genetic variation should be controlled for in future studies [14, 55]. Parity is also associated with socio-environmental factors, and as such, these effects may differ with different access to contraception and childcare. In the absence of these data, we cannot rule out the possibility that the observed relationships between cortical thickness and parenthood are explained by some of these uncollected variables. Future studies should control for these factors, as well as examine the potential effects of grandparenthood and other opportunities for interaction with children during later life. Further, prospective longitudinal studies are needed to draw conclusions about the longevity of these effects; whether our observed relationships are evident across the life-span and reflect effects that have stabilised following the early postpartum period, or whether these effects are specific to late-life parenthood (caring for adult children and potentially grandchildren). Future studies to investigate neurobiological changes across parenting of multiple children in humans are required, as there are currently no such studies in humans at any life-stage [48, 56]. Our findings are the first to show a relationship between number of children and grey matter thickness in the ageing human brain. These preliminary findings should be interpreted in light of their limitations, and we encourage future research to replicate these effects in a prospective longitudinal study.

## Conclusion

This study is the first examination of the relationship between parenthood and the ageing human brain. Our results suggest that there is a modest, but reproducible, relationship between parenthood and cortical thickness in the aged brain. The pattern of results are consistent with published neuroanatomical and behavioural findings in early stages of parenthood, and reveal distributed differences in cortical thickness related to parenthood that are evident beyond the post-partum period. The understanding of the generalised neural changes related to human parenthood is relatively poor, even during pregnancy and the early post-partum period. The neuroscience of parenthood is an important area for further research to identify the critical time periods of parenthood when the brain is highly plastic, and examine longitudinal changes beyond the immediate post-partum months. The nascent field of the neuroscience of parenting has the potential to elucidate the neurobiological and neuroanatomical impact of the human reproductive experience, which may provide unique insights into mechanisms of neuroplasticity.

## Supporting information

S1 File (DOCX)

## References

[1] Stolzenberg DS, Champagne FA. Hormonal and non-hormonal bases of maternal behavior: The role of experience and epigenetic mechanisms. Horm Behav. 2016;77:204–10. 10.1016/j.yhbeh.2015.07.005 26172856

[2] Hillerer KM, Neumann ID, Couillard-Despres S, Aigner L, Slattery DA. Lactation-induced reduction in hippocampal neurogenesis is reversed by repeated stress exposure. Hippocampus. 2014;24(6):673–83. 10.1002/hipo.22258 24615851

[3] Kinsley CH, Amory-Meyer E. Why the maternal brain? J Neuroendocrinol. 2011;23(11):974–83. 10.1111/j.1365-2826.2011.02194.x 21790810

[4] Oatridge A, Holdcroft A, Saeed N, Hajnal JV, Puri BK, Fusi L, et al Change in brain size during and after pregnancy: study in healthy women and women with preeclampsia. American Journal of Neuroradiology. 2002;23(1):19–26. 11827871 PMC7975506

[5] Kim P, Leckman JF, Mayes LC, Feldman R, Wang X, Swain JE. The plasticity of human maternal brain: longitudinal changes in brain anatomy during the early postpartum period. Behav Neurosci. 2010;124(5):695–700. 10.1037/a0020884 20939669 PMC4318549

[6] Kim P, Dufford AJ, Tribble RC. Cortical thickness variation of the maternal brain in the first 6 months postpartum: associations with parental self-efficacy. Brain Structure and Function. 2018;223(7):3267–77. 10.1007/s00429-018-1688-z 29855765 PMC6358213

[7] Hoekzema E, Barba-Muller E, Pozzobon C, Picado M, Lucco F, Garcia-Garcia D, et al Pregnancy leads to long-lasting changes in human brain structure. Nat Neurosci. 2017;20(2):287–96. 10.1038/nn.4458 27991897

[8] Kim P, Rigo P, Mayes LC, Feldman R, Leckman JF, Swain JE. Neural plasticity in fathers of human infants. Soc Neurosci. 2014;9(5):522–35. 10.1080/17470919.2014.933713 24958358 PMC4144350

[9] Lisofsky N, Gallinat J, Lindenberger U, Kuhn S. Postpartal Neural Plasticity of the Maternal Brain: Early Renormalization of Pregnancy-Related Decreases? Neurosignals. 2019;27(1):12–24. 10.33594/000000105 31112016

[10] Gatewood JD, Morgan MD, Eaton M, McNamara IM, Stevens LF, Macbeth AH, et al Motherhood mitigates aging-related decrements in learning and memory and positively affects brain aging in the rat. Brain Res Bull. 2005;66(2):91–8. 10.1016/j.brainresbull.2005.03.016 15982524

[11] Kinsley CH, Madonia L, Gifford GW, Tureski K, Griffin GR, Lowry C, et al Motherhood improves learning and memory. Nature. 1999;402(6758):137–8. 10.1038/45957 10647003

[12] Kinsley CH, Franssen RA, Meyer EA. Reproductive experience may positively adjust the trajectory of senescence. Behavioral Neurobiology of Aging: Springer; 2011 p. 317–45. 10.1007/7854_2011_123 21611905

[13] Kozorovitskiy Y, Hughes M, Lee K, Gould E. Fatherhood affects dendritic spines and vasopressin V1a receptors in the primate prefrontal cortex. Nature neuroscience. 2006;9(9):1094–5. 10.1038/nn1753 16921371

[14] de Lange A-MG, Kaufmann T, van der Meer D, Maglanoc LA, Alnæs D, Moberget T, et al Population-based neuroimaging reveals traces of childbirth in the maternal brain. Proceedings of the National Academy of Sciences. 2019;116(44):22341–6. 10.1073/pnas.1910666116 PMC6825266 31615888

[15] Ning K, Zhao L, Franklin M, Matloff W, Batta I, Arzouni N, et al Parity is associated with cognitive function and brain age in both females and males. Sci Rep. 2020;10(1):6100 10.1038/s41598-020-63014-7 32269255 PMC7142076

[16] Ward SA, Raniga P, Ferris NJ, Woods RL, Storey E, Bailey MJ, et al ASPREE-NEURO study protocol: A randomized controlled trial to determine the effect of low-dose aspirin on cerebral microbleeds, white matter hyperintensities, cognition, and stroke in the healthy elderly. Int J Stroke. 2017;12(1):108–13. 10.1177/1747493016669848 27634976 PMC6764436

[17] Storey AE, Walsh CJ, Quinton RL, Wynne-Edwards KE. Hormonal correlates of paternal responsiveness in new and expectant fathers. Evolution and Human Behavior. 2000;21(2):79–95. 10.1016/s1090-5138(99)00042-2 10785345

[18] McNeil JJ, Woods RL, Nelson MR, Murray AM, Reid CM, Kirpach B, et al Baseline characteristics of participants in the ASPREE (ASPirin in Reducing Events in the Elderly) study. The Journals of Gerontology: Series A. 2017;72(11):1586–93. 10.1093/gerona/glw342 PMC5861878 28329340

[19] Teng E, Chui H. The modified mini-mental state examination (3MS). Can J Psychiatry. 1987;41(2):114–21. 3611032

[20] Katz S, Akpom CA. A measure of primary sociobiological functions. International journal of health services. 1976;6(3):493–508. 10.2190/UURL-2RYU-WRYD-EY3K 133997

[21] Ross TP. The reliability of cluster and switch scores for the Controlled Oral Word Association Test. Archives of Clinical Neuropsychology. 2003;18(2):153–64. 14591467

[22] Benedict RH, Schretlen D, Groninger L, Brandt J. Hopkins Verbal Learning Test–Revised: Normative data and analysis of inter-form and test-retest reliability. The Clinical Neuropsychologist. 1998;12(1):43–55.

[23] Smith A. Symbol Digit Modality Test (SDMT) Manual. Los Angeles: Western Psychological Services 1982.

[24] Troyer AK, Leach L, Strauss E. Aging and response inhibition: Normative data for the Victoria Stroop Test. Aging, Neuropsychology, and Cognition. 2006;13(1):20–35. 10.1080/138255890968187 16766341

[25] Radloff LS. The CES-D scale: A self-report depression scale for research in the general population. Applied psychological measurement. 1977;1(3):385–401.

[26] Dale AM, Fischl B, Sereno MI. Cortical surface-based analysis: I. Segmentation and surface reconstruction. Neuroimage. 1999;9(2):179–94. 10.1006/nimg.1998.0395 9931268

[27] Desikan RS, Ségonne F, Fischl B, Quinn BT, Dickerson BC, Blacker D, et al An automated labeling system for subdividing the human cerebral cortex on MRI scans into gyral based regions of interest. Neuroimage. 2006;31(3):968–80. 10.1016/j.neuroimage.2006.01.021 16530430

[28] Rosen AF, Roalf DR, Ruparel K, Blake J, Seelaus K, Villa LP, et al Quantitative assessment of structural image quality. Neuroimage. 2018;169:407–18. 10.1016/j.neuroimage.2017.12.059 29278774 PMC5856621

[29] Seo SW, Im K, Lee J-M, Kim ST, Ahn HJ, Go SM, et al Effects of demographic factors on cortical thickness in Alzheimer's disease. Neurobiology of aging. 2011;32(2):200–9. 10.1016/j.neurobiolaging.2009.02.004 19321233

[30] Veit R, Kullmann S, Heni M, Machann J, Häring H-U, Fritsche A, et al Reduced cortical thickness associated with visceral fat and BMI. NeuroImage: Clinical. 2014;6:307–11. 25379443 10.1016/j.nicl.2014.09.013 PMC4215386

[31] Luders E, Kurth F, Gingnell M, Engman J, Yong EL, Poromaa IS, et al From baby brain to mommy brain: Widespread gray matter gain after giving birth. Cortex. 2020;126:334–42. 10.1016/j.cortex.2019.12.029 32105976

[32] Swain JE, Lorberbaum JP, Kose S, Strathearn L. Brain basis of early parent-infant interactions: psychology, physiology, and in vivo functional neuroimaging studies. J Child Psychol Psychiatry. 2007;48(3–4):262–87. 10.1111/j.1469-7610.2007.01731.x 17355399 PMC4318551

[33] Köhler S, Black S, Sinden M, Szekely C, Kidron D, Parker J, et al Memory impairments associated with hippocampal versus parahippocampal-gyrus atrophy: an MR volumetry study in Alzheimer’s disease. Neuropsychologia. 1998;36(9):901–14. 10.1016/s0028-3932(98)00017-7 9740363

[34] Burgmans S, van Boxtel M, van den Berg K, Gronenschild E, Jacobs H, Jolles J, et al The posterior parahippocampal gyrus is preferentially affected in age-related memory decline. Neurobiology of aging. 2011;32(9):1572–8. 10.1016/j.neurobiolaging.2009.09.008 19879667

[35] Davies SJ, Lum JA, Skouteris H, Byrne LK, Hayden MJ. Cognitive impairment during pregnancy: a meta-analysis. Med J Aust. 2018;208(1):35–40. 10.5694/mja17.00131 29320671

[36] Buckwalter JG, Buckwalter DK, Bluestein BW, Stanczyk FZ. Pregnancy and postpartum: changes in cognition and mood. Progress in brain research. 133: Elsevier; 2001 p. 303–19. 10.1016/s0079-6123(01)33023-6 11589139

[37] Kinsley CH, Lambert KG. Reproduction-induced neuroplasticity: natural behavioural and neuronal alterations associated with the production and care of offspring. J Neuroendocrinol. 2008;20(4):515–25. 10.1111/j.1365-2826.2008.01667.x 18266940

[38] Wager TD, Davidson ML, Hughes BL, Lindquist MA, Ochsner KN. Prefrontal-subcortical pathways mediating successful emotion regulation. Neuron. 2008;59(6):1037–50. 10.1016/j.neuron.2008.09.006 18817740 PMC2742320

[39] Atzil S, Hendler T, Feldman R. Specifying the neurobiological basis of human attachment: brain, hormones, and behavior in synchronous and intrusive mothers. Neuropsychopharmacology. 2011;36(13):2603–15. 10.1038/npp.2011.172 21881566 PMC3230500

[40] Laurent HK, Ablow JC. A cry in the dark: depressed mothers show reduced neural activation to their own infant’s cry. Social cognitive and affective neuroscience. 2012;7(2):125–34. 10.1093/scan/nsq091 21208990 PMC3277361

[41] Raz N, Ghisletta P, Rodrigue KM, Kennedy KM, Lindenberger U. Trajectories of brain aging in middle-aged and older adults: regional and individual differences. Neuroimage. 2010;51(2):501–11. 10.1016/j.neuroimage.2010.03.020 20298790 PMC2879584

[42] Kinsley CH, Bardi M, Karelina K, Rima B, Christon L, Friedenberg J, et al Motherhood induces and maintains behavioral and neural plasticity across the lifespan in the rat. Arch Sex Behav. 2008;37(1):43–56. 10.1007/s10508-007-9277-x 18074214

[43] Etkin A, Egner T, Kalisch R. Emotional processing in anterior cingulate and medial prefrontal cortex. Trends in cognitive sciences. 2011;15(2):85–93. 10.1016/j.tics.2010.11.004 21167765 PMC3035157

[44] Olson IR, Plotzker A, Ezzyat Y. The enigmatic temporal pole: a review of findings on social and emotional processing. Brain. 2007;130(7):1718–31. 17392317 10.1093/brain/awm052

[45] Lenzi D, Trentini C, Pantano P, Macaluso E, Iacoboni M, Lenzi GL, et al Neural basis of maternal communication and emotional expression processing during infant preverbal stage. Cereb Cortex. 2009;19(5):1124–33. 10.1093/cercor/bhn153 18787229

[46] Henry NJE, McMichael M, Johnson J, DiStasi A, Roland P, Wilford KL, et al RN maternal newborn nursing: review module2016.

[47] Everette A, Fleming D, Higgins T, Tu K, Bardi M, Kinsley C, et al Paternal experience enhances behavioral and neurobiological responsivity associated with affiliative and nurturing responses. International Behavioral Neuroscience Society, Rio de Janeiro. 2007.

[48] De Carli P, Costantini I, Sessa P, Visentin S, Pearson RM, Simonelli A. The expectant social mind: A systematic review of face processing during pregnancy and the effect of depression and anxiety. Neurosci Biobehav Rev. 2019;102:153–71. 10.1016/j.neubiorev.2019.04.013 31055013

[49] Parsons CE, Young KS, Petersen MV, Jegindoe Elmholdt EM, Vuust P, Stein A, et al Duration of motherhood has incremental effects on mothers' neural processing of infant vocal cues: a neuroimaging study of women. Sci Rep. 2017;7(1):1727 10.1038/s41598-017-01776-3 28496095 PMC5431892

[50] Broomhill R, Sharp R. The changing male breadwinner model in Australia: A new gender order? Labour & Industry: a journal of the social and economic relations of work. 2005;16(1):103–27.

[51] Salat DH, Buckner RL, Snyder AZ, Greve DN, Desikan RS, Busa E, et al Thinning of the cerebral cortex in aging. Cerebral cortex. 2004;14(7):721–30. 10.1093/cercor/bhh032 15054051

[52] Carmona S, Martínez‐García M, Paternina‐Die M, Barba‐Müller E, Wierenga LM, Alemán‐Gómez Y, et al Pregnancy and adolescence entail similar neuroanatomical adaptations: a comparative analysis of cerebral morphometric changes. Human brain mapping. 2019;40(7):2143–52. 10.1002/hbm.24513 30663172 PMC6865685

[53] De Lange A-MG, Barth C, Kaufmann TG, Anatürk M, Suri S, Ebmeier KP, et al History of childbirths relates to region-specific brain aging patterns in middle and older-aged women. BioRxiv. 2020. 10.1002/hbm.25152 PMC7555081 32767637

[54] Kim P, Strathearn L, Swain JE. The maternal brain and its plasticity in humans. Horm Behav. 2016;77:113–23. 10.1016/j.yhbeh.2015.08.001 26268151 PMC4724473

[55] Barban N, Jansen R, De Vlaming R, Vaez A, Mandemakers JJ, Tropf FC, et al Genome-wide analysis identifies 12 loci influencing human reproductive behavior. Nature genetics. 2016;48(12):1462–72. 10.1038/ng.3698 27798627 PMC5695684

[56] Maupin AN, Roginiel AC, Rutherford HJ, Mayes LC. A preliminary review of whether prior reproductive experience influences caregiving. New directions for child and adolescent development. 2016;2016(153):73–86. 10.1002/cad.20169 27589499

