# Supplementary material for: Relationship between parenthood and cortical thickness in late adulthood
Source: PLoS One. 2020 Jul 28;15(7):e0236031. doi: 10.1371/journal.pone.0236031 (PMC7386609; doi:10.1371/journal.pone.0236031)
Supplement: S1 File — (DOCX) [file pone.0236031.s001.docx]

**Relationship between Parenthood and Cortical Thickness in Late Adulthood**

Orchard, Ward, Sforazzini, Storey, Egan & Jamadar

**~ Supplementary Material ~**

**Supplementary Methods**

| **S1 Table.** *Participant numbers in the study cohort at 0, 12 and 36 months showing a 15% attrition across three years.* | | | | |
| --- | --- | --- | --- | --- |
|  | | **Time-point 1**  **(0 months)** | **Time-point 2**  **(12 months)** | **Time-point 3**  **(36 months)** |
| N (Males) | 287 | | 271 | 244 |
| N (Females) | | 260 | 236 | 213 |
| N (Total) | | 547 | 507 | 457 |

**
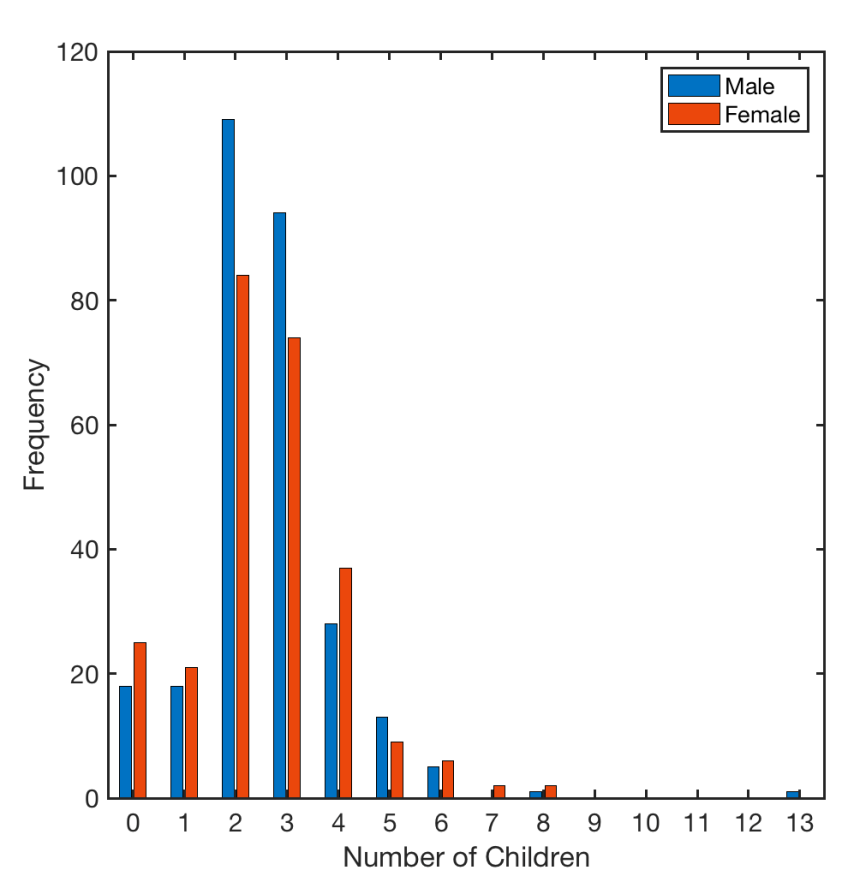
**

**S1 Fig.** *Number of children per participant, split by gender*

**Supplementary Results**

In this section, we report the reproducibility of the results from the main study. In the main manuscript, we report the relationship between parenthood and cortical thickness at baseline, prior to study medication. Here, we report the results at Timepoint 2 and 3, as well as the mean thickness across all three timepoints to determine the reproducibility of the results. Note that sample sizes for each subsequent timepoint are slightly smaller due to study attrition (Supplementary Table 1).

*Hypothesis 1: the relationship between number of children and cortical thickness*. For females, the trend of increased parahippocampal gyrus thickness, and reduced cuneus and pericalcarine sulcus thickness with increasing number of children was maintained at timepoints 2 and 3. Since these trends were reproduced across timepoints, we conclude that these effects, while modest, are likely to be true positives.

**S2 Table.** *Reproducibility of relationship between number of children and cortical thickness for females and males.*

| **Females** | **Region** |  | **Mean** | **Time-point 1**  N= 235 | **Time-point 2**  N=213 | **Time-point 3**  N=191 |
| --- | --- | --- | --- | --- | --- | --- |
|  | R Parahippocampal Gyrus | *r* | 0.19 | 0.15 | 0.16 | 0.22 |
|  |  | *p* | 0.006 | 0.026 | 0.02 | 0.005 |
|  | L Cuneus | *r* | -0.17 | -0.13 | -0.15 | -0.18 |
|  |  | *p* | 0.020 | 0.052 | 0.003 | 0.026 |
|  | L Pericalcarine Sulcus | *r* | -0.19 | -0.11 | -0.14 | -0.15 |
|  |  | *p* | 0.008 | 0.112 | 0.044 | 0.007 |
|  | R Precuneus | *r* | -0.12 | -0.12 | -0.06 | -0.14 |
|  |  | *p* | 0.097 | 0.073 | 0.38 | 0.083 |

*Abbreviations:* H, hemisphere; R, right; L, left; r, Spearman’s Rho; p, p value.

*Hypothesis 2: parents compared to non-parents*: For females, the trend of reduced cortical thickness for mothers compared to non-mothers was retained in timepoints 2 and 3. This suggests that the effect in the middle frontal gyrus and pericalcarine sulcus, while modest, are likely to be true positives. For males, the trend of increased cortical thickness for fathers vs. non-fathers was also retained, but the effect in the temporal pole was notably weaker at timepoints 2 and 3 than timepoint 1. These results suggest that the effect in the temporal pole should be interpreted with some caution. The effect of fatherhood in the anterior cingulate was reproduced, suggesting that it may be a true positive.

**S3 Table.**  *Cortical thickness differences between parents (one child) and non-parents for males and females.*

| **Females** | **Region** |  | **Mean** | **Time-point 1**  N_(Mothers)_ = 20  N(_Non-Mothers)_ = 25 | **Time-point 2**  N_(Mothers)_ = 20  N(_Non-Mothers)_ =23 | **Time-point 3**  N_(Mothers)_= 17  N(_Non-Mothers)_ = 22 |
| --- | --- | --- | --- | --- | --- | --- |
|  | L Caudal Middle Frontal Gyrus (DLPFC) | *d* | -0.87 | -0.93 | -0.60 | -0.86 |
|  |  | *p* | 0.040 | 0.006 | 0.13 | 0.16 |
|  | R Pericalcarine Sulcus | *d* | -0.89 | -0.59 | -0.42 | -0.71 |
|  |  | *p* | 0.032 | 0.066 | 0.29 | 0.21 |
| **Males** | **Region** |  | **Mean** | **Time-point 1**  N_(Fathers)_ = 18  N(_Non-Fathers)_ =17 | **Time-point 2**  N_(Fathers)_ = 17  N(_Non-Fathers)_ =17 | **Time-point 3**  N_(Fathers)_ = 16  N(_Non-Fathers)_ =17 |
|  | L Caudal Anterior Cingulate Cortex | *d* | -1.04 | -0.71 | -0.89 | -1.07 |
|  |  | *p* | 0.054 | 0.047 | 0.052 | 0.14 |
|  | R Temporal Pole | *d* | 0.81 | 1.00 | 0.52 | 0.17 |
|  |  | *p* | 0.11 | 0.007 | 0.24 | 0.78 |

*Abbreviations:* L, left; R, right; d, Cohen’s d; p, p value

| **S3 Table.** *Correlations between cortical thickness and cognition.* | | | | |
| --- | --- | --- | --- | --- |
|  | **Region** | ***r*-value** | ***Uncorrected p*-value** | |
| **COWAT** | L. Temporal Pole  R. Temporal Pole  R. Entorhinal  R. Parahippocampal gyrus | 0.19  0.13  0.14  0.14 | | 0.002  0.037  0.029  0.029 |
| **HVLT** | R. Entorhinal | 0.16 | | 0.012 |
| **SDMT** | L. Bank of the Superior Temporal Sulcus  L. Entorhinal  L. Isthmus Cingulate  L. Lateral Orbitofrontal Gyrus  L. Paracentral Gyrus  L. Precentral Gyrus  L. Superior Frontal Gyrus  L. Superior Temporal Gyrus  L. Supramarginal Gyrus  L. Mean Thickness  R. Caudal Anterior Cingulate  R. Caudal Middle Frontal Gyrus  R. Entorhinal  R. Fusiform gyrus  R. Middle Temporal Gyrus  R. Pericalcarine  R. Precentral Gyrus  R. Superior Temporal Gyrus | 0.13  0.16  0.15  0.13  0.20  0.13  0.13  0.16  0.13  0.14  -0.14  0.14  0.19  0.17  0.15  0.13  0.13  0.20 | | 0.043  0.009  0.015  0.033  0.001  0.036  0.037  0.008  0.035  0.021  0.022  0.031  0.003  0.007  0.013  0.036  0.036  0.001 |
| **CES-D** | L. Frontal Pole  R. Caudal Anterior Cingulate | 0.13  0.13 | | 0.045  0.040 |
| **STROOP** | L. Entorhinal  L. Middle Temporal Gyrus  L. Paracentral Gyrus  L. Precentral Gyrus  L. Precuneus  L. Superior Temporal Gyrus  R. Bank of the Superior Temporal Sulcus R. Caudal Middle Frontal Gyrus  R. Entorhinal  R. Middle Temporal Gyrus  R. Pericalcarine  R. Superior Temporal Gyrus | -0.12  -0.13  -0.19  -0.21  -0.13  -0.16  -0.13  -0.14  -0.18  -0.13  -0.14  -0.20 | | 0.047  0.031  0.003  <0.001  0.031  0.012  0.036  0.022  0.003  0.031  0.025  0.001 |
